# Aducanumab and Its Effects on Tau Pathology: Is This the Turning Point of Amyloid Hypothesis?

**DOI:** 10.3390/ijms23042011

**Published:** 2022-02-11

**Authors:** Serena Silvestro, Andrea Valeri, Emanuela Mazzon

**Affiliations:** IRCCS Centro Neurolesi “Bonino-Pulejo”, Via Provinciale Palermo, Contrada Casazza, 98124 Messina, Italy; serena.silvestro@irccsme.it (S.S.); andrea.valeri@irccsme.it (A.V.)

**Keywords:** Alzheimer’s disease, beta-amyloid, monoclonal antibody, immunization, aducanumab, Tau pathology

## Abstract

Alzheimer’s disease (AD) is a neurodegenerative disorder affecting millions of people around the world. The two main pathological mechanisms underlying the disease are beta-amyloid (Aβ) plaques and intracellular neurofibrillary tangles (NFTs) of Tau proteins in the brain. Their reduction has been associated with slowing of cognitive decline and disease progression. Several antibodies aimed to target Aβ or Tau in order to represent hope for millions of patients, but only a small number managed to be selected to participate in clinical trials. Aducanumab is a monoclonal antibody recently approved by the Food and Drug Administration (FDA), which, targeting (Aβ) oligomers and fibrils, was able to reduce Aβ accumulation and slow the progression of cognitive impairment. It was also claimed to have an effect on the second hallmark of AD, decreasing the level of phospho-Tau evaluated in cerebrospinal fluid (CSF) and by positron emission tomography (PET). This evidence may represent a turning point in the development of AD-efficient drugs.

## 1. Introduction

Alzheimer’s Disease (AD) is the most common form of dementia and represents a great burden for patients, families, and public healthcare. With more than 50 million affected people all around the world [1], AD remains a great challenge for researchers and pharmaceutical companies. This devastating and deadly disease shows itself through impairment in daily life activities, memory loss, and behavioral changes until the patient becomes completely dependent on their family or caregivers. Despite the fact that Dr. Alois Alzheimer first described this disease at the beginning of 1900 [2], its causes are still not known: factors such as genetics, environment, and lifestyle play together and make the general picture so confusing that today, in 2022, humanity still does not have a cure for AD, and the main risk factor remains the inevitable aging process. The main hallmarks of AD are extracellular deposition of beta-amyloid (Aβ) and neurofibrillary tangles (NFTs) made from hyperphosphorylated Tau (p-Tau) protein, along with neuronal loss [3,4,5]. For a long time, the scientific community was divided between the so-called “Tauists” and “βaptists”, depending on which of the two main hallmarks was thought to be sufficient and necessary for the disease developing [6], assuming that the presence of one on them is dependent on the other. While the debate proceeded between Tauist, βaptists, and “agnostics”, many believed that both hallmarks were equally relevant or that some other process led to AD. Pharmaceutical companies tried to address patients’ needs, developing and testing a long list of drugs, especially monoclonal antibodies [7]. In June 2021, the Food and Drug Administration (FDA) approved the monoclonal antibody aducanumab as an immunotherapeutic approach against AD [8]. Aducanumab (BIIB037) is a monoclonal antibody born from molecular cloning of human memory B cell collections reactive against aggregated Aβ, both in the form of soluble oligomers and insoluble fibrils [9]. In the results of the clinical trials, the sponsor Biogen claimed a decrease in Aβ plaques and p-Tau, assessed by CSF analyses and positron emission tomography (PET) imaging [10]. Is this the evidence that the scientific community is waiting for to better understand the mechanism behind AD? Could this give an indication of the future direction of pharmacological treatment for AD patients?

The first aim of this review is to analyze the pre-clinical evidence regarding a possible connection between Aβ reduction and Tau reduction and the other way around, followed by an analysis of AD monoclonal antibody clinical trials results in which one hallmark influences the other. The second aim is indeed to understand if the evidence is replicated in clinical trials and if treatment with anti-Aβ or anti-Tau is favored as a possible future for AD research.

## 2. Methods

**Figure 1:**
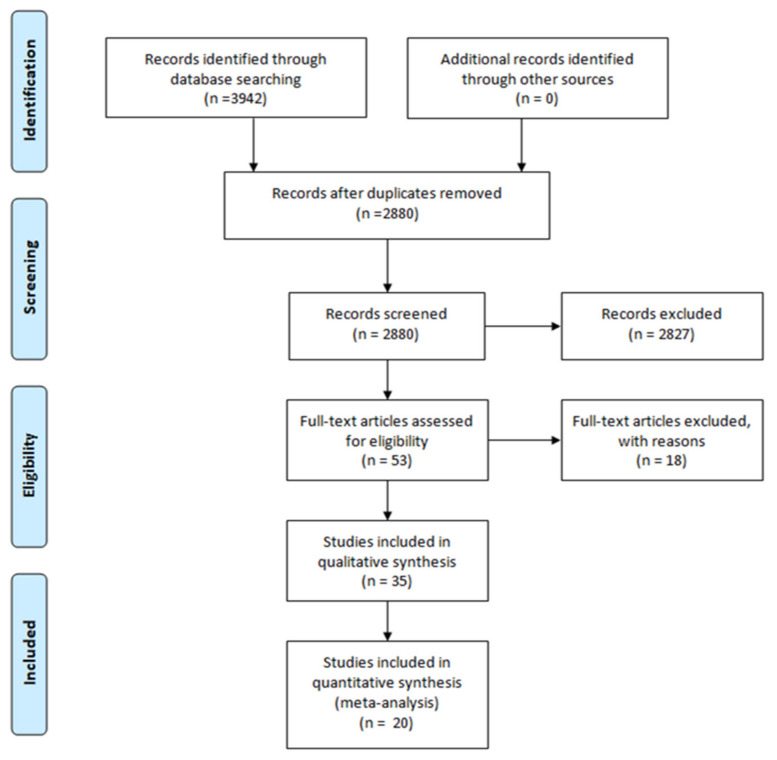
The Prisma flow diagram shows the methodology for selecting the articles included in this review. Duplicate articles were excluded from the total of the studies recorded. Instead, articles that evaluated the role of the immunization against Aβ or Tau, which has the effect of reducing Tau pathology and Aβ, respectively, were considered. The PRISMA Statement is published in [11].

In order to obtain the material for this review, we considered English articles published between 2012 and 2022, which demonstrated a dependence of one hallmark on the other. We used the keywords “Tau antibody”, “amyloid beta reduction”, “amyloid beta antibody”, and “Tau reduction” in PubMed. The keyword “amyloid beta” was written as such or as “Aβ” in order to include articles that also contain the abbreviation. The articles taken into consideration were the ones which described immunization against Aβ or Tau, which has the effect of reducing Tau pathology and Aβ, respectively. In vivo studies and clinical trials’ evidence were included in the analyses. We excluded reviews and not relevant publications, as the Prisma flow diagram reports in Figure 1. The PRISMA Statement is published in [11].

## 3. Alzheimer’s Disease: Aβ and Tau against Neurons

AD is a progressive neurodegenerative disease and the main cause of dementia worldwide. The progression is usually slow and, apart from the familiar form, normally has a late onset. Furthermore, there is no cure and no possibility of regression: once the neurodegeneration has started, there is no way to stop it or revert it. In most cases, AD occurs on a sporadic base (SAD) with late-onset, but in a small percentage of cases (<0.5%), AD has an early onset due to genetic mutations, known as familial AD (FAD). In 70% of cases, FAD is caused by mutations in three genes: the amyloid precursor protein (APP), presenilin 1 (PSEN1), and presenilin 2 (PSEN2). Mutations of these genes provoke a change in the production or processing of Aβ, corroborating the amyloid hypothesis [12,13]. The PSEN1 and PSEN2 genes encode, respectively, for presenilin 1 and presenilin 2 enzymes, belonging to the protease class A that regulates the functions of the γ-secretase enzyme [14]. A SAD may be due to a polymorphism in the *APOE* gene encoding apolipoprotein E (APOE), a protein involved in lipid metabolism [15,16]. The *APOE* gene has three variants, ε2, ε3, and ε4, the latter being responsible for the conversion of Aβ into fibrillar form and its deposition. Furthermore, APOE4 also appears to influence Aβ clearance [17]. Novel genetic approaches using next-generation sequencing have identified a number of low-frequency genes that confer a relatively high risk of developing AD [18]. Recent evidence shows that a healthy lifestyle, such as regular exercise and a balanced diet, can reduce the risk of developing AD. It was observed that physical activity stimulates neurogenesis, synaptic plasticity, and neurotrophin production [19].

Different hypotheses have divided the scientific community for a long time regarding the trigger and the mechanism behind the development of the disease.

### 3.1. βAptits Bell: The Amyloid Cascade Hypothesis

At the beginning of the disease, there is Aβ accumulation. The formation of these misfolded protein plaques results in a gain of toxic functions, provoking the formation of neurofibrillary tangles of Tau protein, vascular dysfunctions, and inflammation, leading to neuron death in turn, ends in dementia [20]. In physiological conditions, APP is involved in brain homeostasis [21]. In particular, following the amyloidogenic pathway, APP is processed by α-secretase generating sAPPα, a soluble neuroprotective molecule [22,23]. The amyloidogenic pathway is the main process of Aβ biogenesis in healthy subjects, while, on the contrary, APP is processed by β- and γ-secretase in pathological conditions [24]. β-secretase makes a first cut generating sAPP-β, a 99-amino acid chain, which is then processed by the γ-secretase to generate a peptide of 40 (Aβ_1–40_) and 42 amino acids (Aβ_1–42_) [25,26]. Among the two peptides, Aβ_1–42_ shows high insolubility and tends to fibrillate. Indeed, it represents the most hydrophobic, amyloidogenic, and toxic peptide [27,28]. Aβ monomers undergo structural changes leading to their aggregation into oligomeric form, which can then form fibrils that deposit into the amyloid plaques. These plaques generally tend to accumulate in the isocortex and only later in the subcortical areas, generating the inflammatory process responsible for irreversible damage to neurons [29,30]. It is thought that an excessive production or reduced clearance of Aβ promotes self-assembly in amyloid-plaque, which is a hallmark in pathological samples of AD patients. For decades, cytotoxicity has been attributed predominantly to Aβ amyloid fibrils [31]; however, it has recently been shown that soluble Aβ oligomers are much more harmful [32,33].

### 3.2. Tauists Bell: Tau as Cause and Marker of Neurodegeneration

The second hallmark of AD is represented by NFTs. They consist of coupled helical filaments, which are formed after Tau hyperphosphorylation in the cell body of neurons. The Tau protein is normally responsible for stabilizing microtubules and vesicular transport. In neuronal cells, microtubules play important roles in conferring neuronal structure, axon transport, and neuronal plasticity [34]. In physiological conditions, the Tau protein is regulated by a perfect balance between the phosphorylated and dephosphorylated state, but in a pathological state, excessive phosphorylation induces NFT formation, responsible for the destabilization and neuron death [35]. The p-Tau protein is first identified in the magnocellular nuclei of the basal forebrain, the oral nuclei of the raphe system of the brainstem, and the locus coeruleus [36]. NFTs appear initially in the superficial layer of the transentorhinal cortex, the entorhinal cortex, and subsequently, in the hippocampus, the middle temporal gyrus, the superior temporal gyrus, and the remaining cortex [36,37]. Like Aβ, p-Tau can also accumulate in the dendritic spines at the beginning of the pathological process, altering postsynaptic transmission. In this way, p-Tau can spread trans-synaptically between connected neurons and induce the pathogenesis of AD [38,39,40]. It is noteworthy that the degeneration induced by NFTs in the basal proecephalon partly causes damage to the cholinergic neurons and the diffuse loss of presynaptic cholinergic innervation in the neocortical and limbic zone [41].

### 3.3. The “Agnostic” Bell: Different Causes for One Disease

Even though the scientists who do not belong to the βaptist or Tauist faction do not deny the importance of Aβ and NFTs in the progression of AD, other players are thought to have their part in the development of the disease. One of these is acetylcholine (ACh) because of its role in cognition: memory, attention, and learning, seems to be influenced by the cholinergic system in the brain, so its impairment could be involved in the development of AD. It is also important to notice that, despite the fact that Aβ deposits can influence synaptic transmission and lead to their degeneration, the presence of Aβ is not always correlated with the severity of the disease [42]. Alterations to the Wnt pathway can cause both the accumulation of NFTs and synaptic loss due to the activation of microglia. Hence, neuroinflammation is another possible cause of neurodegeneration, and it is not always correlated with the presence of Aβ [43]. Recently, the brain–gut axis gained significant attention and investigation regarding its possible role in brain diseases, so a theory about the release of toxic substances from the intestine, or neuroinflammatory cytokines, and even direct microbial infection was developed as alternative causes of AD [44].

## 4. Therapies for Alzheimer’s Disease

To date, no treatments are yet available that are capable of modifying the underlying pathology or the course of the disease. Despite the growing number of AD patients, currently, only four drugs and one combined treatment are approved by the FDA and authorized by the European Union [45], which are donepezil, galantamine, and rivastigmine, three acetylcholinesterase (AChE) inhibitors, and memantine, an N-methyl-D-aspartate receptor antagonist. AChE inhibitors act to increase the availability of acetylcholine and inhibit its breakdown in synapses. These drugs are well tolerated, especially when introduced in low doses and gradually titrated. In AD patients, AChE inhibitors have been reported to have beneficial effects in both mild and severe forms of AD. Obviously, dosage, escalation times, and route of administration vary according to the patient, allowing personalized treatment options [46]. Memantine is often used as a first-line drug in severe AD, and it is also used in the management of AD disorders. Thanks to its low affinity toward the N-methyl-D-aspartate receptor, memantine reduces the excitatory neurotoxicity of L-glutamate without altering physiological functions. Treatment with memantine appears to improve cognitive impairment and reduce the functional decline in moderate to severe AD patients [47]. The fifth type of treatment, approved in 2014, involves the use of combination therapy using donepezil and memantine, at fixed-dose [48]. The drugs’ combination is a valid therapeutic option that allows modulating different therapeutic targets. Recently, the use of disease-modifying therapies (DMTs) enabled simultaneously targeting amyloid plaques formation, Tau accumulation, or other pathological processes. Combination therapies can also be used to hit the same pathological mechanism. For example, a monoclonal antibody directed against the formation of amyloid plaques can be used in combination with a β-secretase (BACE 1) inhibitor in order to prevent plaque re-accumulation [49]. The use of BACE1 inhibitors is not effective in AD patients whose Aβ accumulation is already high enough to induce dementia, and they seem not only to lack efficacy when administered alone but worsen the condition of patients. This was probably due to the fact that Aβ also plays a physiological role, and its aberrant accumulation might give rise to the disease, not its presence per se. This may also explain why similar devastating effects on cognition are not replicated in patients who underwent treatment with antibodies against Aβ [50]. Another therapeutic strategy involves the use of humanized monoclonal antibodies that activate an immunomodulatory response against the Aβ peptide, increasing its clearance [51]. Phase III studies, which were expected to be completed between 2019/2021, are evaluating the effects of these monoclonal antibodies, such as gantenerumab and crenezumab [52,53,54]. Unfortunately for patients, the crenezumab trials on the sporadic form of AD were discontinued since they were unlikely to meet the expected endpoints, while efficacy and safety were evaluated in patients who have a risk of developing AD due to autosomal dominant mutations [55].

Tau aggregation inhibitors are another therapeutic strategy aimed at preventing or reducing Tau aggregation [56,57]. However, the drug Leuco-methylthioninium bishydromethanesulfonate (LMTM) did not benefit patients with mild/moderate AD [58]. Masitinib, a selective tyrosine kinase inhibitor, acts as a mast cell antagonist by modulating pro-inflammatory mediators. Additionally to standard therapies, this agent could reduce the neuroinflammation involved in AD pathogenesis [59,60]. Therefore, given the failures of many anti-AD agents, add-on therapies to standard drugs involving inhibition of BACE1, Tau aggregation, and Aβ immunization could provide encouraging results. This supports research toward new therapeutic strategies.

Several ongoing trials are also evaluating the effects of symptomatic treatments in addition to standard therapy. Depression, anxiety, agitation, and aggression are neuropsychiatric symptoms that often occur in AD patients. Currently, there is no approved drug therapy to treat these symptoms associated with AD. However, second-generation antidepressants and antipsychotics are used as off-label to improve neuropsychiatric symptoms. The efficacy of this therapy has shown mixed results. Currently, a promising antipsychotic drug that could be useful in the future is primavanserin: it acts on the 5-HT_2A_ receptor as an inverse agonist, and it has already demonstrated its efficacy in the treatment of Parkinson’s Disease-derived or Alzheimer’s Disease-derived psychosis. The subsequent clinical trial HARMONY was designed in order to understand the efficacy and safety of primavanserin in the treatment of symptoms such as delusion or hallucination, common in various types of dementia [61].

### Anti-Amyloid Antibody Therapies, Aducanumab and Aβ-Tau Decrease

In AD patients, the soluble neurotoxic oligomers’ formation generated by monomeric Aβ molecules is followed by insoluble polymeric Aβ aggregates’ formation, which tends to accumulate in amyloid plaques [62]. According to the amyloid hypothesis, this mechanism is responsible for the neurodegenerative process in AD, leading to harmful effects on neuron function and synaptic plasticity, with consequent learning and memory deficits [63]. It is known that the dimerization of monomeric Aβ is responsible for the formation of aggregates. Indeed, in AD patients, the presence of Aβ dimers is correlated with the clinical manifestation of the disease [64]. Therefore, anti-amyloid therapy represents a promising strategy for the management of AD because it prevents cerebral amyloidogenesis through pathological Aβ oligomerization inhibition [51]. Most of the treatments in use are anti-amyloid therapies. Among them, immunotherapy is the most elaborate treatment, which can be defined as active immunization when the immune system is stimulated to produce its own antibodies or passive immunization when exogenous antibodies are used [65,66]. Active immunotherapy has the advantage of stimulating a long-term immune reaction, providing a short treatment with limited costs. On the other hand, especially in elderly subjects, active immunotherapy may be absent and induce adverse effects, even for prolonged periods [67,68,69,70]. Conversely, passive immunization, through the use of monoclonal antibodies, promotes a high antibody titer, and the interruption of the treatment allows resolving the adverse events. However, the disadvantage of using monoclonal antibodies is the need for repeated administration and the high cost [71]. It seems that monoclonal antibodies have the ability to cross the blood–brain barrier (BBB) and penetrate the parenchyma, where they inhibit toxic Aβ oligomerization or induce plaque clearance by microglia [72]. Additionally, these antibodies have also been shown to have anti-inflammatory effects, capable of reducing the production of pro-inflammatory cytokines [73]. In the last years, several monoclonal antibodies have been studied aimed at the elimination of Aβ, many of which have also moved into clinical trials.

In June 2021, the FDA approved aducanumab as a treatment for Alzheimer’s Disease after the sponsor Biogen obtained and analyzed the complete data from the clinical trials. Before discussing the finding, which correlates Aβ removal with Tau decrease, it is important to understand what aducanumab is and its mechanism of action.

Aducanumab is a recombinant human immunoglobulin (IgG1) monoclonal antibody that arises from a collection of blood lymphocytes from healthy elderly donors who do not have signs of cognitive impairment or slow decline. This antibody has been shown to be effective in reducing brain Aβ plaques [52]. Compared to other antibodies, aducanumab establishes a unique link with the peptide Aβ, which prompts researchers to explore its structural kinetics and interaction links with the peptide. Aducanumab binds to the Glu3 and Asp7 residues of the Aβ peptide, and it shows a low binding affinity toward the monomeric form of Aβ [9]. Its strength lies in the capacity of the binding aggregation of Aβ, which includes oligomers and fibrils [74]. This binding is capable of recruiting microglia into the site of Aβ aggregates, so it can accomplish phagocytosis of the deposits and clear the brain from these toxic substances before they are able to damage the neurons: once the microglia is activated and has reached the site of Aβ plaques, it isolates the core and prevents the formation of further oligomers. Moreover, the binding between Aβ oligomers and metabotropic receptors is prevented by the one between microglia and Aβ oligomers, avoiding the depolarization of the membrane and neuronal calcium overload [52]. Last, but not less important, aducanumab’s own the capacity of binding to fibrils, thus decreasing the secondary nucleation of Aβ aggregation [75]. The aggregation of Aβ is a multi-stage process that involves a primary nucleation step, in which the monomeric aggregates of Aβ are formed, followed by a phase of growth of the monomeric aggregates by adding monomers. This phase precedes the secondary nucleation step, characterized by the multiplication of monomeric aggregates on the surface of fibrils already formed [76]. Aducanumab decreases the formation of the oligomeric aggregates, directly inhibiting the latter molecular reaction. Additionally, comparing the action of aducanumab with the one of chaperone Brichos, a selective inhibitor of secondary nucleation [77], it was observed that this monoclonal antibody exhibited a similar kinetic profile [75].

As Biogen Senior Vice President and Head of the Neurodegeneration Development Unit declared to the FDA Peripheral and Central Nervous System Drug Advisory Committee on 6 November 2020, in the analyses of the data of their clinical studies emerged the evidence that Aβ immunotherapy has the effect, in a small subset of patients, of reducing the amount of phospho-Tau, highlighted by CSF and Tau PET imaging of the medial temporal lobe [78]. The decrease was also significantly correlated with clinical outcomes since phospho-Tau is linked to cognitive decline [79]. This was declared the first report of the evidence of Aβ immunotherapy capacity to influence Tau pathophysiology, highlighted by two different and independent modalities [10].

## 5. Anti-Aβ and Anti-Tau Preclinical Studies: Searching for the Link between Hallmarks

Intravenous immunoglobulins (IVIs) were thought to be useful in the treatment of AD for the presence of polyclonal antibodies against Aβ because they could be found, even with a low titer, in the blood of patients affected by AD. IVIs were tested in triple transgenic mice, which are affected both by Aβ deposits and NFTs, in order to understand if this approach could have any effect on the behavior and memory of the animals. Indeed, a reduction in the Aβ_40_/Aβ_42_ ratio was observed, along with a reduction in Aβ*56 soluble oligomers species, suggested to be responsible for learning and memory impairment. It seems that IVIs could prevent the aggregation of the oligomers and thus affect the degradation and clearance in order to ameliorate the condition of AD at early stages. Even though no variation of Tau was found, previous studies connected Aβ*56 with soluble pathological Tau and this, in turn, correlated with the development of AD in its early stages. IVIs also had immunomodulatory effects and reduced the expression of fractalkine (CX3CR1) receptors. Behavioral deficits of the mouse model were attenuated, suggesting that immunotherapy could be a way to pursue to fight AD [80]. Unfortunately, immunoglobulin infusion failed to improve cognition in the participants of the Phase III clinical trial [81].

The concept of preventing the aggregation of Aβ and decreasing the amount of the already-present plaques is in line with the choice of Biogen to include in their studies patients at an early stage of AD, which, of course, needed to present Aβ plaques in their brains.

A computational study suggested exactly that, so an Aβ immunotherapy could be efficient only at the early stage of the disease and might require combination with other therapies. However, the design of the model included a possible effect on Tau phosphorylation based on previous evidence, but it is important to mention that it does not directly assume Aβ to have an effect on Tau. The model predicts Aβ reduction to be linked to p-Tau reduction. According to the simulation, Aβ immunization will lead to a reduction in plaques while only reducing the soluble fraction of Aβ to a small extent. These can have an effect on glycogen synthase kinase 3 beta (GSK3β), which, in turn, decreases the amount of p-Tau, and this seems to remain constant long after immunization. It is important, though, to notice that the model predicts the decrease in p-Tau but not of the NFT amount. Neither seems to be efficient against cellular stress, despite the fact that the reactive oxygen species (ROS) number will be reduced after plaque clearance. This highlights the importance of acting on the early stage of the disease in order to prevent NFT accumulation and ROS production, to maintain a low baseline of stress in the brain of the patients. ROS can indeed have an effect on p53 protein, involved in several pathways, including apoptosis. An important limitation of this model is the fact that it is based on a cellular model for a period of 12 days, and it is, of course, a simplification of the real complex mechanism behind AD that is yet not fully understood [82].

Shifting from computational to a mouse model, the 3xTg-AD model was used to test a vaccination against Aβ. Mice developed antibodies against Aβ and showed a T-helper 2 (Th2)-polarized anti-Aβ antibody response, avoiding the recruiting of Th1, which, together with Th2, provoked the aseptic meningoencephalitis in previous clinical trials of another vaccine. Moreover, in this situation, mice were immunized before Aβ appearance, supporting the idea that a kind of prevention of AD, rather than a cure, could be efficient in fighting the disease. This vaccination, through the decrease in both soluble and insoluble forms of Aβ, decreases both intracellular and extracellular amounts of Aβ, and it has the important effect of reducing soluble and insoluble Tau levels in the brain. As the last step, cognitive performances were evaluated, and vaccinated mice performed better than non-vaccinated ones [83].

Aducanumab itself, during the preclinical tests, proved to decrease Aβ levels in its chimeric form, binding with more affinity to the fibrillary form rather than monomeric one. The idea to target Aβ seeds, even before the plaques started to form, was the basis of an experiment using the chimeric version of two antibodies, aducanumab and gantenerumab, and the murine version of crenezumab and donanemab. Results indicated that it is possible to target Aβ seeds before the detection of Aβ deposits, reducing in such a way the deposits at 6 months. An important finding lies in the evidence that chimeric aducanumab and mE8 (donanemab) were able to recognize Aβ aggregates, while the others also bind to monomers and failed to reduce Aβ seeds, and this could be explained with a sort of “distraction mechanism” from the monomers. Furthermore, treatment with chimeric aducanumab was also observed to reduce p-Tau-induced neuritic changes triggered by the amylogen process and changes in microglia [84].

The decrease in pathological Aβ in mice had the effect of increasing the density of synapses and showed amelioration in the mitochondrial lesions, two important features of AD pathology. It was then no surprise to see an improvement in cognitive performance. In addition, in this model, the level of p-Tau was decreased, and it was not possible to address the effect on NFTs since they usually appeared later than 6 months of age [85]. However, the improvement in cognitive performance was seen after the decrease in Aβ_42_, followed by the decrease in p-Tau, as seen in aducanumab clinical trials results.

DNA Aβ_42_ immunization proved to be efficient in reducing the number of plaques in the brain of the AD mouse model but was also efficient in fighting Tau pathology. It seemed that this immunization could induce a non-inflammatory immune response, so no inflammatory cytokines were produced, and there was no proliferation of T-cells when high levels of IgG2a/c were detected. This activates and guides the microglia in removing the Aβ excess. DNA immunization also reduced the amount of Aβ_42_ to a better extent than direct immunization with Aβ_42_ peptides, indicating how antibodies generated from DNA immunization can detect different Aβ species. Immunization has, moreover, the effect of reducing the protein level of ERK1/2, as well as GSK3β protein levels, and this is important because ERK2 can phosphorylate a large amount of residue in Tau protein. Since Aβ oligomers activate both Tau kinases and caspases, their reduction also prevents the phosphorylation and aggregation of Tau, resulting in a reduction in total Tau and Tau aggregation [86].

Another strategy of immunization is represented by the blocking of the APP cleavage site of BACE1, so BACE-APP interaction is prevented as the generation of Aβ toxic species. Indeed the use of this monoclonal antibody in a triple transgenic mouse model has the effect of reducing intracellular Aβ deposits, leading to a decrease also in total Tau and p-Tau levels, probably due to lower GSK3β activity. As previously described, both Aβ and Tau play their role in damaging the mitochondria at different levels of mitochondrial complexes, so this antibody was also able to prevent mitochondrial dysfunctions. Another important effect was the decrease in microglia and astrocytes activation, thus preventing the inflammatory response that is thought to be a key event in neurodegeneration. Cognitive functions were significantly better after immunization, probably due to the increase in synaptophysin, an indicator of synaptic plasticity [87].

There are several hypotheses concerning the mechanism of Aβ clearance from the blood, and one of these is the so-called “peripheral sink”, where a gradient of Aβ is created through the BBB and Aβ is then cleared from the blood. Using this mechanism, a monoclonal antibody against Aβ was conjugated with PEGilate liposomes, in order to avoid the probable loss of material due to the crossing of BBB. The conjugate antibody was administered first to human plasma, then cells, and then in double transgenic mice. It maintained its capacity to bind to Aβ monomers and oligomers in plasma, reducing the uptake from the capillaries and improving the clearance by macrophages. In mice, the conjugated antibody administration induced a drop in Aβ blood concentration and also decreased the level of p-Tau, as well as reducing the amount of activated glia. However, important information came from the evidence that this conjugated antibody was more effective in old mice than in adult mice, probably due to the worse condition of the BBB in old mice, which permits a better antibody translocation into the brain, or the different type of circulating Aβ in the blood of the adults [88].

This latter evidence raises the question if it is really necessary to intervene in the early stage of pathology to see any effect. A study using a monoclonal antibody directed against β-sheet conformation proteins showed a decreased amount of Aβ and p-Tau also in mice in the late stage of the disease. The unique feature of this antibody is to bind protein with β-sheet-dominant structures, the only common feature between Aβ and Tau pathology. The cognitive improvements were reported to be significant with no difference in locomotor activity between the treated and non-treated groups. Even though the antibody was able to bind both Aβ and Tau, the reduction was seen in the amount of Aβ_40_/Aβ_42_ and aggregated soluble species, but not in NFTs, despite the decrease in oligomeric and extracellular p-Tau, which is thought to be responsible for the spread of the Tau pathology in a “prion-like” manner [89]. These results indicated that a reduction in both Aβ and p-Tau is necessary to see any effects on cognitive performances, but also suggested that a late intervention might not be useless.

The monoclonal antibody bapinezumab was modified in order to avoid the side effect of microglia over activation and administered to the triple transgenic mice at the late stage of the disease. In the hippocampus, the reduction in Aβ_40_ and Aβ_42_ and also a reduction in Tau was achieved. This result was important because the treatment was performed when the pathology was well established in the model. The mechanism underlying this clearance is probably the peripheral sink [90].

A combined approach was tested in order to target both the pathological hallmarks, and it was carried out by vaccination against Aβ and Tau pathology. The chosen mouse model, the T5x mouse line, developed efficient antibodies and this led to a reduction in both the pathological hallmarks of AD. However, there were data also from single vaccination against Aβ and a single vaccination against Tau: the first one led to a decrease in Tau. Instead, the second vaccination failed to a decreased Aβ_42_ amount, supporting the idea that Aβ can have a direct effect on Tau and the other way around is not happening, or is less prominent [91].

Furthermore, from the anti-Tau point of view, some efforts were made to understand if targeting the protein which makes the NFTs could lead to some improvement in AD pathology.

Triple transgenic mice were immunized against Tau using a Tau vaccine and its effects on Tau pathology, Aβ, and behavioral tests were analyzed. The decrease in Tau pathology was also accompanied by a reduction in Aβ deposits and, most importantly, these benefits remained even 16 months after the last injection. It was supposed that neurons affected by Tau pathology are prone to generate more Aβ, whose deposits are located close to these “sick” neurons and their synapses. This could explain why Aβ plaques are often surrounded by neurons that are positive for Tau aggregates. No improvement in behavioral tests was found, mainly because no defects were assessed by the cognitive tests performed, probably because the triple transgenic mice cognitive impairment required different analyses to be clear [92].

A similar result was obtained by another set of anti-Tau antibodies on the same mouse model and, along with a decrease in p-Tau, total Tau, and a trend to also decrease Aβ, improvements were also found in reference memory after the Morris Water Maze test. In particular, reference memory improvement was seen in the mice treated with the antibody which can target the normal N-terminal of Tau protein. The mechanism suggested is that Tau immunization can activate the complement which in turn leads Aβ to proteolysis, but it could also be involved a reduction in APP synthesis. Another important finding was that is possible to also reduce p-Tau when the disease is at its late stage [93].

Targeting the correct part of protein Tau seems crucial in the design of the antibodies for Tau-therapy. Another experiment demonstrated that targeting the 20–22 kDa NH2-terminal Tau fragment is necessary to decrease both pathological Tau and the metabolism of APP, preventing the production of Aβ. The synaptic plasticity was also preserved in the mouse models chosen for the experiment, one of which was the triple transgenic one. Indeed there was also an improvement in the learning and memory skills associated with the hippocampus. The antibody administration was not followed by the activation of microglia, so neuroinflammation was avoided and the neutralization of the toxic species was directly mediated by the antibody, which also works to block the uptake of the toxic substances by the neurons [94].

**Figure 2:**
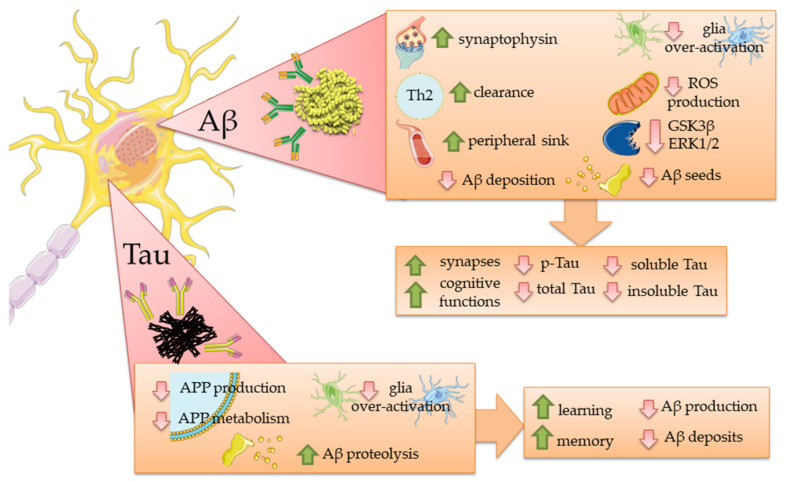
Effects of antibodies against Aβ or Tau on the second AD hallmark emerged from preclinical studies. The main effects of the antibodies in the cellular processes are reported in the first quadrant, followed by the consequences on Tau, in case of Aβ antibodies, or Aβ, in case of Tau antibodies. The image was created using the image bank of Servier Medical Art (Available online: http://smart.servier.com/, accessed on 20 January 2022), licensed under a Creative Commons Attribution 3.0 Unported License (Available online: https://creativecommons.org/licenses/by/3.0/, accessed on 20 January 2022). Aβ: beta-amyloid; APP: amyloid precursor protein; ERK1/2: extracellular signal-regulated kinases 1 and 2; GSK3β: Glycogen synthase kinase 3 beta; p-Tau: phospho-Tau; ROS: Reactive Oxygen Species.

Figure 2 resumes the mechanisms that are supposed to influence the AD hallmarks after the administration of antibodies against Aβ or Tau. Table 1 resumes the mechanisms and the outcomes of the previously described preclinical tests.

## 6. Clinical Evidence: Are Preclinical Findings Replicated in Humans?

During the clinical trial phases, aducanumab efficacy was tested on patients with Aβ deposits at the early/mild stage of AD. The safety and tolerability were investigated in the phase I trial (NCT01397539), where one of the adverse effects was amyloid-related imaging abnormalities (ARIA) with areas of hyperintensity on fluid-attenuation inversion recovery magnetic resonance imaging (MRI), reflecting parenchymal edema or sulcal effusion (ARIA-E). ARIA-E seems to be a common adverse effect in Aβ immunotherapy. However, the treatment showed safety and tolerability up to a dose of 30 mg/kg and results confirmed the low binding affinity of aducanumab to soluble monomeric Aβ [95]. As the PRIME study (NCT01677572) suggested, aducanumab’s effects on the clinical signs of AD are dose-dependent and this will be confirmed by the quantification of brain Aβ load over time in response to the treatment [52,96]. The two main phase III trials were called ENGAGE (NCT02477800) and EMERGE (NCT02484547), and their results were the ones taken into consideration by the FDA to approve or reject the drug. The decrease in Aβ plaques was determined by PET, and it was significant, while the decrease in p-Tau using the same technique was found in a small subset of patients, similar to the CSF measurements. What was important was the correlation between Aβ reduction, CSF p-Tau, and the clinical outcomes of EMERGE, the trial which met the primary outcome. The cognitive decline was slowed, and this was assessed using different measures, such as Clinical Dementia Rating Sum of Boxes (CDR-sb), Mini-Mental State Examination, Alzheimer’s Disease Assessment Scale-cognitive subscale, and the Alzheimer’s Disease Cooperative Study Activities of Daily Living (ADCS ADL). Caregiver distress, which was, of course, reduced to a great extent (84%) was taken into consideration [79]. It is important to mention that ENGAGE and EMERGE were prematurely interrupted for futility [97], and the next data were first analyzed by the sponsor and then presented to FDA, which resulted in the approval, which gave rise to criticisms [98]. The accelerated approval by the FDA was based on a surrogate endpoint, which is the reduction in Aβ plaques observed in both studies (EMERGE and ENGAGE). However, the accelerated approval of the drug is accompanied by the requirement to perform a “post-marketing” trial aimed at generating additional efficacy and safety data for aducanumab [99].

Aducanumab is, of course, not the only antibody against Aβ which reached the clinical trial phase. Other antibodies managed to be tested in human patients and some of them also exert an effect on Tau protein. It is important to note that these trials were carried out before aducanumab’s, so their data could have been useful to optimize the protocol for recruiting and administration of the next trials.

Gantenerumab (RO4909832, RG1450) is raised against Aβ and its mechanism of Aβ clearance involves phagocytosis mediated by the Fc receptor. During the SCarlet RoAD study (NCT01224106; WN25203) patients with prodromal AD were treated with the antibody, but the trial did not meet expectations. However, from a biological point of view, there was a reduction in amyloid load after PET measuring and also a reduction in Tau in CSF, assessed by the amount of p-Tau and t-Tau, a marker of neuronal axon degeneration. All these effects were dose-dependent. Unfortunately, the trial was stopped early for futility, so it is not possible to understand if a higher dose would have shown some clinical improvement, even if curiously the group of fast-progressing AD patients showed a numerical difference in cognitive decline, not replicated in the slow-progressor [100]. The clinical trial involving gantenerumab and solanezumab on early symptomatic or asymptomatic SAD patients, despite demonstrating how gantenerumab can decrease the main hallmarks of AD, resulted in no slowing of clinical decline [101]. However, the biological evidence seemed to correlate with the reduction in Aβ with the reduction in Tau.

Bapineuzumab, an antibody against the N-terminal part of Aβ, was tested in patients with mild/moderate AD in two different trials (NCT00575055 and NCT00574132), and unfortunately failed to improve clinical outcomes. Interesting findings involved the correlation between Aβ decrease and p-Tau decrease in the CSF because it was found only in *APOE ε4* carrier patients and not in the non-carriers [102]. Further analyses showed an increased risk of developing ARIA-E and increased copies of *APOE ε4* and with increasing the Bapinezumab dose. The decrease in CSF p-Tau and t-Tau was bigger in patients who experienced ARIA-E, along with a greater reduction in Aβ. However, these also correlated with an enlargement of the ventricles and reduction in hippocampal volume [103]. Bapineuzumab trials were the first to show a correlation between Aβ reduction and t-Tau and p-Tau in CSF (NCT00112073) and since t-Tau is correlated with a poor prognosis in brain disorders, as well as the level of p-Tau seems to reflect the NFTs formation in the brain, further investigation was carried out [104]. In this study, Aβ and Tau reduction did not seem very important, since there was no clinical improvement in both groups of carriers and non-carriers.

Donanemab is a humanized IgG1 antibody directed at an N-terminal pyroglutamate Aβ epitope that is present only in established plaques. The treatment reduced Aβ plaque levels but did not seem to change Tau levels after the first analyses. Additional analyses, however, suggested a reduction in Tau accumulation in the frontal and temporal regions of the brain of treated patients. As previously mentioned, ARIA-E incidence was more in APOE-ε4 carriers. Regarding cognitive impairment, donanemab patients seemed to have a slower cognitive and clinical decline than placebo-treated patients [105]. Apart from APOE-ε4 carrier evidence, the results are quite similar to aducanumab’s, maybe giving some hope that this is a good way to follow.

AN-1792 trials suggested a role of microglia behind the accumulation of Tau and Aβ. AN-1792 was the first active immunotherapy for AD and the immunization was carried out using a synthetic Aβ peptide. After immunization, a decrease in Aβ and p-Tau was found, and there was no correlation between the level of Tau and microglia. The chronic activation of microglia may lead to hyperphosphorylation of Tau due to the release of pro-inflammatory cytokines and could also correlate with Aβ. Normally there is an inverse correlation between Aβ and microglia activation markers, with the aim of limiting the neuronal damage, but the immunization seems to alter this correlation in order to induce Aβ clearance. It is important, though, to note that microglia seems focused on Aβ removal after immunization [106]. The strategy of directing microglia in Aβ removal is intriguing, but could represent a double-edge strategy, since it has to be strictly controlled in order to not give rise to an indiscriminate clearance.

From an anti-Tau treatment point of view, the news is not better than the anti-amyloid ones. Unfortunately for the patients who are waiting for a solution to this devastating disease, a series of failures in anti-Tau clinical trials is bad news [107]. Different antibodies reached the clinical trials phase (Table 2) and some of them are still ongoing. Surprisingly, there is no evidence that anti-Tau treatment will decrease Aβ in human patients.

## 7. Conclusions

Despite the preclinical evidence that both Tau and Aβ can influence each other if targeted for immunotherapy, only a small percentage of the clinical trials confirmed this data. The recent approval of aducanumab might have shifted the market scale in favor of an anti-Aβ therapy in order to reach some concrete results for patients, but its influence on Tau levels might also have opened an important window in the field of AD research: can we act on Tau pathology by preventing Aβ accumulation? Assuming that Aβ and Tau are interplayers in AD pathology, could it be true that Tau pathology is attenuated enough by Aβ removal that cognitive impairment will be delayed? Aducanumab data point in this direction, but more data and refinement in treatment protocols are necessary before answering these questions. Will we ever reach the point that AD could be fully prevented by just one injection of antibodies?

## Figures and Tables

**Table 1:** Preclinical tests where one of the main AD hallmark proved to influence the other.

Type of Immunization	Primary Target(s)	Outcome	Possible Mechanism	Ref.
Intravenous immunoglobulins	Aβ	Aβ_40_/Aβ_42_ reduction Aβ*56 soluble oligomers reduction Attenuation behavioral deficits Reduction in CX3CR1 receptor expression	Prevention of oligomers aggregation	[80]
Aβ immunization (computational model)	Aβ	Prediction of: p-Tau reduction Aβ plaques reduction	Reduction in Aβ plaques Decrease in GSK3β activity	[82]
Vaccination	Aβ	Th2-polarized anti-Aβ antibody response Decrease in intra- and extra-cellular Aβ Reducing soluble and insoluble Tau Improving cognitive performances	Decrease in soluble and insoluble Aβ	[83]
cmAducanumab cmGantenerumab murine-Crenezumab murine-Donanemab	Aβ	Decrease in Aβ seeding Reduced p-Tau, induced neuritic changes Reduced microglia changes	Limit amylogen process Binding to fibrillary Aβ	[84]
A8	Aβ	Increase in synaptic density Ameliorate mitochondrial lesions Improving cognitive performances Decrease in p-Tau	Decrease in pathological Aβ	[85]
DNA Aβ_42_ immunization	Aβ	Removing Aβ excess Reduce in total Tau Reduce Tau aggregation	Microglia guidance to Aβ deposits Reduce ERK1/2 protein level Reduce GSK3β protein level	[86]
BBS1	APP-BACE1 interaction	Reducing intracellular Aβ Decrease in total Tau Decrease in p-Tau Prevention of mitochondrial dysfunction Prevention of inflammation Improvement in cognitive functions	Prevention of Aβ generation Decrease in GSK3β activity Decrease in glia activation Increase in synaptophysin	[87]
STAB-mAb immunoPEGliposomes	Aβ	Reduce capillary uptake of Aβ monomers and oligomers Improve macrophages clearance Drop in Aβ blood concentration Decrease in p-Tau level Reduce glia activation	Bind capacity to Aβ monomers and oligomers Peripheral sink mechanism	[88]
GW-23B7	β-sheet proteins	Aβ_40_/Aβ_42_ reduction Aggregated soluble species reduction Oligomeric and extracellular p-Tau reduction	Binding to β-sheets proteins	[89]
Modified Bapinezumab	Aβ	Aβ_40_/Aβ_42_ reduction in hippocampus Reduction in Tau pathology	Peripheral sink mechanism	[90]
Vaccination	Aβ and Tau Single Aβ Single Tau	Aβ and Tau reduction Reduction in Tau after single Aβ vaccination	Generation of high affinity antibodies	[91]
Vaccination	Tau	Aβ decrease Decrease in Tau pathology	Decrease in Aβ production by neurons with Tau pathology	[92]
43D and 77E9	Tau	Decrease in p-Tau Decrease in total Tau Trend in Aβ decreasing Improvement in reference memory	Activation of complement for Aβ proteolysis Reduction APP synthesis	[93]
12A12	Tau	Decrease in pathological Tau Decrease in APP metabolism Preservation of synaptic plasticity Improvement in learning and memory hippocampus-associated No microglia activation	Targeting 20–22 kDa NH2-terminal Tau fragment	[94]

Aβ: beta-amyloid; APP: amyloid precursor protein; cm: chimeric; BACE1: beta-secretase 1; CX3CR1: fractalkine receptor; ERK1/2: extracellular signal-regulated kinases 1 and 2; GSK3β: Glycogen synthase kinase 3 beta; p-Tau: phospho-Tau; Th2: T-helper cells.

**Table 2:** Clinical evidence of antibodies’ effects on AD hallmarks. The table shows the origin and different epitopes of Aβ recognized from the different tested substance and their efficacy.

Substance	Origin	Aβ Epitope	Effects	Ref.
Aducanumab	Human IgG1	A.A. 3-7	Decrease in Aβ Decrease p-Tau in CSF and PET	[79]
Bapineuzumab	Humanized IgG1	A.A. 1-5	Reduction in fibrillar Aβ p-Tau decrease in CSF Correlation Aβ/Tau reduction in APOE ε4	[103,104]
Gantenerumab	Humanized IgG1	A.A. 3-12, 18-27	Reduction in Aβ Reduction p-Tau and t-Tau in CSF	[100]
Donanemab	Humanized IgG1	N-terminal pyroglutamate Aβ	Reduction in Aβ plaques Reduction in Tau accumulation in frontal and temporal lobe	[105]
AN-1792	Synthetic full-length Aβ peptide, QS-21 adjuvant	Aβ N-terminus	Aβ clearance by microglia p-Tau reduction	[106]

Aβ: amyloid-β; A.A.: amino acid; AD: Alzheimer’s disease; CSF: cerebrospinal fluid; Ig: immunoglobulin; p-Tau: phospho-Tau.

## Data Availability

Not applicable.
